# Variations in Circulating Levels of Angiopoietin-2 Over Time Are Predictive of Ramucirumab–Paclitaxel Therapy Outcome in Advanced Gastric Cancer: Results of Prospective Study

**DOI:** 10.3389/fonc.2022.862116

**Published:** 2022-04-06

**Authors:** Rosalba D’Alessandro, Maria Grazia Refolo, Annalisa Schirizzi, Giampiero De Leonardis, Rossella Donghia, Vito Guerra, Gianluigi Giannelli, Ivan Roberto Lolli, Maria Maddalena Laterza, Ferdinando De Vita, Caterina Messa, Claudio Lotesoriere

**Affiliations:** ^1^ Laboratory of Experimental Oncology, National Institute of Gastroenterology - IRCCS “Saverio de Bellis”, Castellana Grotte, Italy; ^2^ Data Science Unit, National Institute of Gastroenterology - IRCCS “Saverio de Bellis”, Castellana Grotte, Italy; ^3^ Scientific Direction, National Institute of Gastroenterology - IRCCS “Saverio de Bellis”, Castellana Grotte, Italy; ^4^ Medical Oncology Unit, National Institute of Gastroenterology - IRCCS “Saverio de Bellis”, Castellana Grotte, Italy; ^5^ Complex Operating Unit Oncologia, Local Health Authority Napoli 2 Nord, P.O. “S.M. delle Grazie”, Naples, Italy; ^6^ Division of Medical Oncology, Department of Precision Medicine, School of Medicine, University of Study of Campania “Luigi Vanvitelli”, Naples, Italy

**Keywords:** angiogenesis, gastric cancer, target therapy, biomarkers, cancer progression

## Abstract

The combination of paclitaxel and ramucirumab is the second-line therapy of choice in the treatment of advanced gastric cancer. To date, no biomarkers are available in gastric cancer to predict the outcome of antiangiogenic therapy. The present prospective study included 35 patients undergoing second-line therapy with ramucirumab and paclitaxel. Serum samples were systematically collected from the beginning of therapy and at each cycle until disease progression. Multiplex analysis of a panel of angiogenic factors identified markers for which the changes at defined time intervals were significantly different in patients with progression-free survival ≤3 (Rapid Progression Group) compared to those with progression-free survival >3 (Control Disease Group). Comparative analysis revealed significantly different results in the two groups of patients for VEGFC and Angiopoietin-2, both involved in angiogenesis and lymphangiogenesis. VEGFC increased in the progressive-disease group, while it decreased in the control-disease group. This decrease persisted beyond the third cycle, and it was statistically significant compared to the basal level in patients with longer progression-free survival. Angiopoietin-2 decreased significantly after 2 months of therapy. At progression time, there was a significant increase in VEGFC and Angiopoietin-2, suggesting the activation pathways counteracting the blockade of VEGFR2 by ramucirumab. Overall results showed that a greater change in VEGFC and Angiopoietin-2 levels measured at the beginning of the third cycle of therapy corresponded to a lower risk of progression and thus to longer progression-free survival.

**Graphical Abstract:**
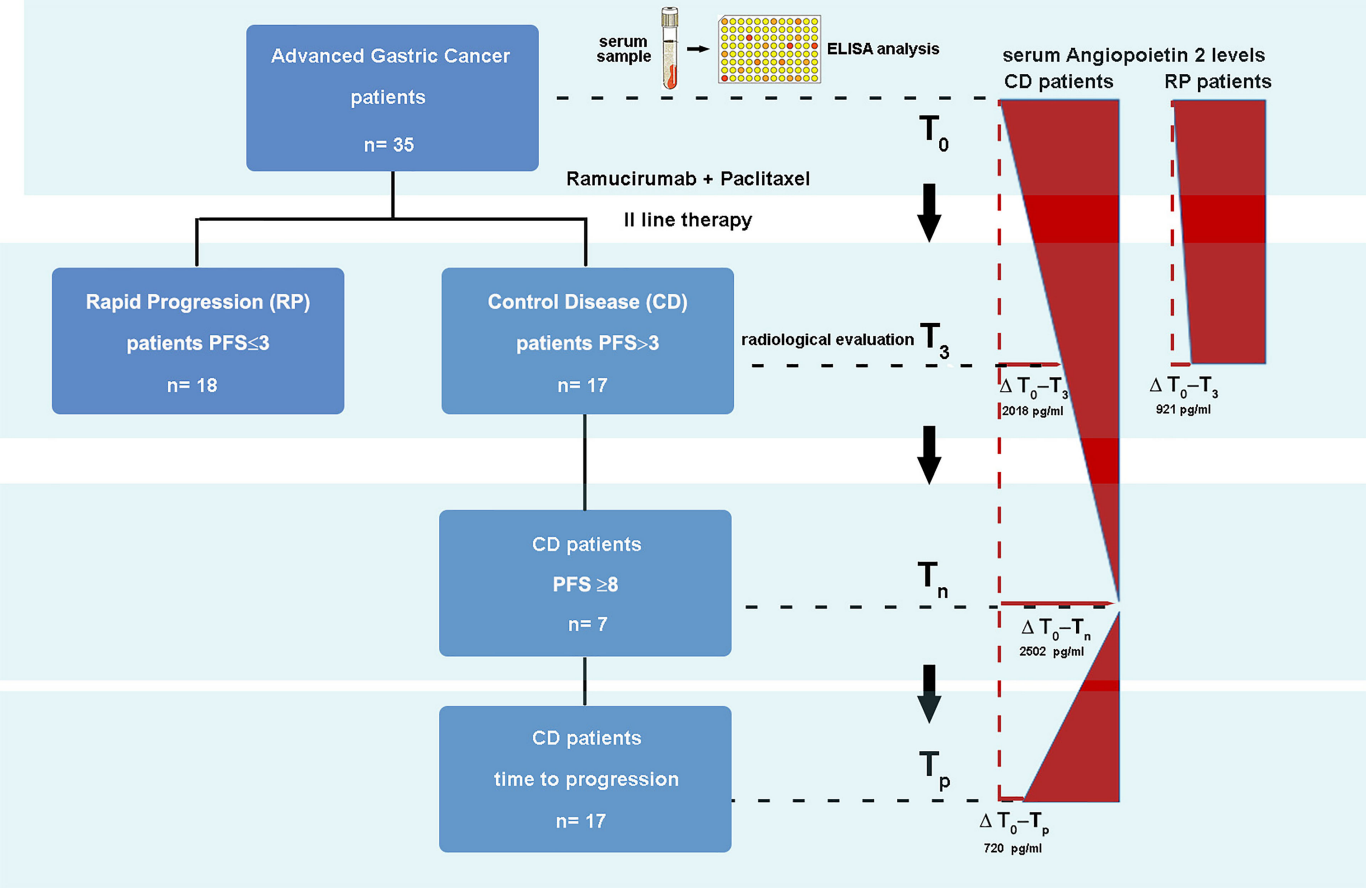


## Introduction

Gastric cancer (GC) currently remains a global health burden, as it ranks as the fifth most common cancer and the fourth leading cause of cancer-related death worldwide. In 2020, more than 1 million new cases were diagnosed globally, and nearly 760,000 deaths occurred (1). The incidence of GC is the highest in Eastern Europe, Eastern Asia, and South America. In Western countries, 80% of patients are diagnosed with unresectable advanced-stage disease or develop a recurrence within 5 years of curative-intent surgery. Thus, the prognosis of advanced GC remains poor with a 5-year survival rate of <30% for all stages and <4% for metastatic disease (2, 3).

Evidence highlights that GC is characterized by a close interdependence between molecular subtype and the angiogenic and immune profile of the tumor microenvironment. As in other solid tumors, several cytokines and growth factors play a dual detrimental role in the tumor microenvironment, as they promote both tumor angiogenesis and immunosuppression. Vascular endothelial growth factors (VEGFs) are the most prevalent and potent promoters of angiogenesis. VEGF family members are involved at different levels in the regulation of the cancer-immunity cycle, producing substantial changes that ultimately contribute to creating a microenvironment that allows the tumor to evade immune surveillance (4, 5).

The actions of VEGFs (VEGFA, VEGFC, and VEGFD) on endothelial cells (ECs) are mediated primarily through the binding and activation of VEGFR-2 [6]. VEGFD and VEGFC mainly bind VEGFR3 involved in lymphangiogenesis (6, 7). Although VEGFC and VEGFD have a high degree of homology, they have different functionalities. VEGFD has a stronger angiogenic potential than VEGFC, which predominantly binds VEGFR3 and acts mainly in the lymphatic system (8, 9). The recent finding of the production of different VEGF ligands and receptors (VEGFRs) in epithelial cancer cells suggests a direct role for these ligands and their receptors in the autocrine control of some biological processes in cancer cells (10–12). The expression levels of VEGFs and VEGFRs in GC correlate with disease prognosis (13). These data represent the scientific rationale for targeting the VEGF pathway in patients with GC.

Ramucirumab, a fully human immunoglobulin IgG1 monoclonal antibody targeting VEGFR-2, is the first antiangiogenic agent to demonstrate activity for advanced GC in a second-line setting. In two pivotal phase III double-blind, placebo-controlled trials, ramucirumab was showed to significantly improve survival when used for therapy either alone (5.2 vs. 3.8 months, hazard ratio (HR) = 0.776, p = 0.047) or combined with paclitaxel (PTX) (9.6 vs. 7.4 months, HR = 0.807, p = 0.017) (14, 15). In the phase III RAINBOW trial, ramucirumab plus PTX improved progression-free survival (PFS) by 1.5 months (median 4.4 vs. 2.9; HR = 0.635, p > 0.0001), with increased response rate (28% vs. 16%, p = 0.0001) and disease control rate (80% vs. 64%, p > 0.0001) as well (15). Real-world data have been shown to support the efficacy and safety of ramucirumab also in daily clinical practice (16).

Although retrospective studies considered VEGF and its receptors as possible biomarkers in gastric carcinoma, the importance of prospective studies evaluating changes in these factors during therapy was underlined (17–21). Furthermore, in a recent study, Van Cutsem and colleagues have investigated a panel of angiogenic markers in patients from the RAINBOW cohort and highlighted some pharmacodynamic and prognostic relationships (22). However, to date, no reliable biomarkers have been identified to select those patients who more likely will benefit from ramucirumab treatment.

The aim of this prospective study is to investigate circulating angiogenic biomarkers in patients with GC undergoing second-line treatment with ramucirumab and PTX.

## Materials and Methods

### Study Design, Serum Sample Collection, and Analysis

The current study provided the enrollment of patients with advanced GC undergoing a second treatment line with ramucirumab and PTX. In this prospective analysis, sera from 35 patients collected before starting therapy and at the first infusion of each cycle were considered. The study was approved by the ethics committee (prot. n°139/c.e. 28-06-2017). Patients provided written informed consent for the collection of blood samples for biomarker analysis. ELISA analysis was performed on serum samples corresponding to basal level (T_0_), the second cycle of therapy (T_2_), the third cycle of therapy (T_3_), and time of radiological and clinical disease progression (T_p_). Regarding VEGFC, Angiopoietin-2 and VEGFR3 were also considered the sixth (T_6_) and ninth cycles of therapy (T_9_). The comparative analysis presented in this study considered times T_0_, T_3_, T_6_, and T_p_. In the analysis, two different groups of patients were distinguished, based on the clinical evaluation after 3 months of treatment: patients who presented disease progression and patients who presented disease control (partial response or stable disease). A total of 13 angiogenetic molecules were analyzed in the serum using two different panels, according to the manufacturer’s instructions with a multiplex bead suspension array kit using Bio-PlexMagPIXSuspension Array System. In panel 1 EGF, Angiopoietin-2 (Ang2), PLGF, VEGFC, VEGFD, FGF2, and VEGFA were analyzed, and in panel 2, there were PDGF, sTIE-2, sEGFR, sVEGFR1, sVEGFR3, and sVEGFR2. These analytes were split into two panels to prevent cross-interferences between beads during the analysis. Each serum sample was analyzed in duplicate, and mean factor concentrations were reported in pg/ml. The serum levels of the aforementioned analytes were dosed beforehand and in different phases of the treatment, in order to associate the variations in their expression with clinical outcomes. Furthermore, linear regression analysis was performed to identify any significant association among VEGF, its receptor, and the other investigated molecules. These results were correlated with the clinical data of each patient.

### Biomarker Detection

In view of interesting results obtained from multiplex beads suspension array analysis, for three of the 13 angiogenetic molecules, a uniplex ELISA was performed to better investigate previous data. VEGFC and Ang2 were quantified using ELISA Kits—QuantikineQuicKit ELISA (R&D Systems, Minneapolis, MN, USA), and sVEGFR3 was dosed using VEGF Receptor 3/FLT4 Human ELISA Kit Invitrogen (Thermo Fisher Scientific, Waltham, MA, USA) according to the manufacturer’s instructions.

### Statistical Analysis

The primary objective of our analysis was to evaluate the relationship between disease progression and single factors involved in the advanced GC.

Patients’ characteristics were reported as mean ± SD (M ± SD) for continuous variables and as frequencies and percentages (%) for categorical variables.

The normal distribution of quantitative variables was tested using the Kolmogorov–Smirnov test.

The variations of single factor measured on individual patients were calculated as differences among baseline time and subsequent evaluation time, and for testing the variations between basal values and those in subsequent times, the sign test was used.

For comparisons of single parameters between the groups as Control and Rapid Progression, the Wilcoxon rank-sum (Mann–Whitney) test was used for continuous variables, so long as the variables were not distributed normally.

Linear regression was used to evaluate the association of individual markers on VEGA, sVEGFR2, Ang2, and VEGFC, where the R-squared is expressed as the goodness-of-fit measure for linear regression models because this statistic indicates the percentage of the variance in the dependent variable that the independent variables explain collectively.

The variation of VEGFC, VEGFR3, and Ang2 between basal time and the third cycle of therapy was divided into tertiles. For studying the time between entry to a study and a subsequent event, such as the progression of the disease, the Cox model was used. The Cox model is a statistical technique for exploring the relationship between the disease progression of a patient and several explanatory variables, and it allows us to estimate the hazard (or risk) of progression for an individual, given its prognostic variables measured as categorical. The Cox proportional hazard model was fitted to the data, and the proportional hazard assumption was evaluated by means of the Schoenfeld residuals test (SRT). Model fitting was evaluated by means of the Akaike information criterion (AIC) and Bayesian information criterion (BIC). Risk estimators were expressed as HR and 95% CI. When testing the null hypothesis of no association, the probability level of error at two tails was 0.05. All the statistical computations were made using STATA (StataCorp. 2021. Stata Statistical Software: Release 17. College Station, TX: StataCorp LLC).

## Results

In the present study, 35 patients with advanced GC undergoing second-line therapy based on ramucirumab and PTX were enrolled. The population was divided into two groups based on the type of response estimated from the first radiological evaluation. In the Control Disease (CD) group, 17 patients who presented response disease or stable disease were included, whereas in the Progression Disease (PD) group, 18 patients were included. In **Table 1**, the population characteristics were reported. The median PFS was of 2.8 and 8.8 months for the PD and CD groups, respectively.

**Table 1:** Clinicopathological features of enrolled patients (n = 35).

Patient features		Category of patients	
		PD^1^	CD^1^
**Enrolled patients**		18	17
**Age, mean (years)**		62	67
**Gender**	*Male*	12	12
	*Female*	6	5
***Tumor features***			
**Location**	*Gastroesophageal Junction*	6	3
	*Fundus of stomach*	1	3
	*Gastric body*	8	6
	*Antrum of stomach*	4	7
	*Whole stomach*	1	/
**Pathological type**	*Intestinal*	4	4
	*Diffuse*	12	13
	*Mixed*	2	/
**Pathological differentiation**	*High differentiation*	1	/
	*Medium differentiation*	3	3
	*Poor differentiation or undifferentiation*	14	14
**HER2 status**	*Positive*	2	1
	*Negative*	16	16
**Primary tumor present**	*Yes*	7	3
	*No*	11	14
**Peritoneal metastasis**	*Yes*	9	9
	*No*	9	8
**Number of metastatic organs**	*0–2*	15	15
	*≥3*	3	2
**Second line of treatment PFS (months)**		2.8	8.8

PFS, progression-free survival. ^1^CD, control disease; PD, progression disease (refer to the first radiological evaluation).

The detection of circulating angiogenic biomarkers levels was performed in multiplex or uniplex array at predefined timing during treatment, as described in the *Materials and Methods*. Comparing the two groups of patients, basal levels of VEGFA, VEGFC, PLGF, and Ang2 in the CD group were higher than those in the PD group (**Table 2**).

**Table 2:** Differences between serum basal levels of biomarkers in control disease group and progression disease group.

*Biomarkers	Basal levels (T_0_)		p^^^
	CD patients (n = 17)	PD patients (n = 18)	
**VEGFC^#^**	6,391.62 ± 3,855.81	5,379.77 ± 2,247.34	0.50
**Ang2^#^**	3,519.88 ± 1,491.77	3,183.22 ± 1,530.03	0.55
**PLGF**	4.43 ± 8.10	2.16 ± 1.63	0.29
**VEGFD**	260.00 ± 122.43	278.80 ± 264.54	0.36
**VEGFA**	179.71 ± 119.77	162.87 ± 104.50	0.73
**sVEGFR1**	32.71 ± 76.77	9.83 ± 3.84	0.21
**sVEGFR2**	2,119.51 ± 772.96	2,660.58 ± 961.85	0.12
**sVEGFR3^#^**	23,256.74 ± 10,117.86	30,979.99 ± 14,826.57	0.19
**sTie2**	2,591.33 ± 1,158.65	2,902.55 ± 1,313.44	0.39

CD, control disease; PD, progression disease (refer to the first radiological evaluation).

^*^Concentration pg/ml, mean ± SD.

^^^Wilcoxon rank-sum (Mann–Whitney) test.

^#^Detected with uniplex ELISA.

On the other hand, the basal levels of VEGFR2, VEGFR3, and sTie2 receptors were lower in the CD group in comparison to the levels of the same receptors detected in the group of patients with progressive disease. The differences, as shown in **Table 2**, were not statistically significant, although they were approaching significance for VEGFR2 (p = 1.12) and VEGFR3 (p = 1.19) receptors.

The mean values of serum levels of each marker were compared between the two groups at time T_3_ versus time T_0_ (**Tables 3A**, **B**). At time T_3_, a significant increase in the levels of the VEGFA, VEGFD, and PLGF ligands was observed with respect to basal levels in both groups. The results for the VEGFC ligand differed between the two groups of patients. In particular, there was a decrease in VEGFC levels in patients within the CD group and an increase in those with the progressive disease when T_3_ and T_0_ were compared (**Tables 3A**, **B**). Considering VEGF receptor levels, a notable decrease was detected in both groups of patients, and the reduction was significant in the case of VEGFR3. In addition, a significant decrease in the Ang2 factor and its receptor sTie2 was detected in both groups when T_3_ and T_0_ were compared (**Table 3**). The analysis of serum levels of other angiogenic factors investigated, including EGF, FGF2, PDGF, and sEGFR, revealed no significant differences in expression levels during treatments in all patients examined (data not shown).

**Table 3A:** Trend of serological biomarkers at the first radiological evaluation on patients with control (**A**) and progression disease (**B**).

*Biomarkers	CD patients (n = 17)		p^^^
	Basal levels (T_0_)	3° Cycle (T_3_)	
**VEGFC^#^**	6,391.62 ± 3,855.81	5,801.32 ± 2,855.72	1.00
**Ang2^#^**	3,519.88 ± 1,491.77	1,501.46 ± 679.81	<0.0001
**PLGF**	4.43 ± 8.10	34.30 ± 30.22	0.0003
**VEGFD**	260.00 ± 122.43	524.51 ± 248.90	<0.0001
**VEGFA**	179.71 ± 119.77	420.09 ± 238.92	0.0003
**sVEGFR1**	32.71 ± 76.77	15.98 ± 26.64	0.63
**sVEGFR2**	2,119.51 ± 772.96	2,009.90 ± 863.59	0.33
**VEGFR3^#^**	23,256.74 ± 10,117.86	3,118.64 ± 2,714.44	<0.0001
**sTie2**	2,591.33 ± 1,158.65	2,005.41 ± 831.52	0.01

**Table 3B:** 

*Biomarkers	PD patients (n = 18)		p^^^
	Basal levels (T_0_)	3° Cycle (T_3_)	
**VEGFC^#^**	5,379.77 ± 2,247.34	7,066.78 ± 4,122.82	0.24
**Ang2^#^**	3,183.22 ± 1,530.03	2,262.26 ± 1,629.11	0.007
**PLGF**	2.16 ± 1.63	35.92 ± 24.96	<0.0001
**VEGFD**	278.80 ± 264.54	460.38 ± 295.60	0.0003
**VEGFA**	162.87 ± 104.50	453.80 ± 203.49	<0.0001
**sVEGFR1**	9.83 ± 3.84	12.27 ± 9.33	0.33
**sVEGFR2**	2,660.58 ± 961.85	2,324.60 ± 1,371.55	0.14
**VEGFR3^#^**	30,979.99 ± 14,826.57	6,081.25 ± 4,102.31	<0.0001
**sTie2**	2,902.55 ± 1,313.44	2,361.33 ± 1,094.16	0.05

CD, control disease; PD, progression disease (refer to the first radiological evaluation).

^*^Concentration pg/ml, mean ± SD.

^^^Sign test.

^#^Detected with uniplex ELISA.

Interestingly, the comparative analysis of the T_0_–T_3_ deltas (ΔT_0_–T_3_) for each analyte in the two groups of patients revealed that the ΔT_0_–T_3_ for VEGFC was positive in the CD group (where the factor decreased by 9.2%) and negative in the PD group (where the factor increased by 31%). Furthermore, the ΔT_0_–T_3_ for Ang2 was significantly greater (p = 0.05) in CD patients than in the PD patients (**Table S1**). Patients within the CD group were followed up until the time of progression (T_p_).

Comparing the serum levels of the investigated ligands at time T_3_ with those at T_p_, a further increase in the VEGFA and PLGF levels was detected; moreover, this increase was statistically significant for VEGFD (p = 0.0002) (**Table 4**). Similar results were obtained for VEGFC and Ang2. In the case of Ang2, the increase at the time of progression in comparison with T_3_ was significant (p = 0.0005) (**Table 4**). In addition, the comparative analysis of receptor levels at time T_3_ versus time T_p_ revealed an increase in both VEGFRs and Tie2 receptor levels (**Table 4**).

**Table 4:** Trend of serological biomarkers from the first radiological evaluation to time of progression.

*Biomarkers	CD patients (n = 16)^§^		p^^^
	3° Cycle (T_3_)	time of progression (>T_p_)
**VEGFC^#^**	5,865.57 ± 2,936.65	6,741.09 ± 4,434.30	0.80
**Ang2^#^**	1,519.55 ± 697.87	2,792.62 ± 1,067.43	0.0005
**PLGF**	35.18 ± 30.99	54.71 ± 51.41	0.58
**VEGFD**	495.33 ± 225.04	704.95 ± 302.45	0.0002
**VEGFA**	423.70 ± 246.28	566.17 ± 339.50	0.58
**sVEGFR1**	16.17 ± 27.50	38.60 ± 101.97	0.27
**sVEGFR2**	2,063.73 ± 861.95	3,492.69 ± 5,275.96	0.58
**VEGFR3^#^**	3,193.42 ± 2,785.32	4,232.80 ± 3,834.45	0.45
**sTie2**	2,075.69 ± 804.96	2,569.63 ± 2,227.84	0.58

CD, control disease.

^*^Concentration pg/ml, mean ± SD.

^^^Sign test.

^§^One patient undergoing therapy.

^#^Detected with a uniplex ELISA.

**Figure 1:**
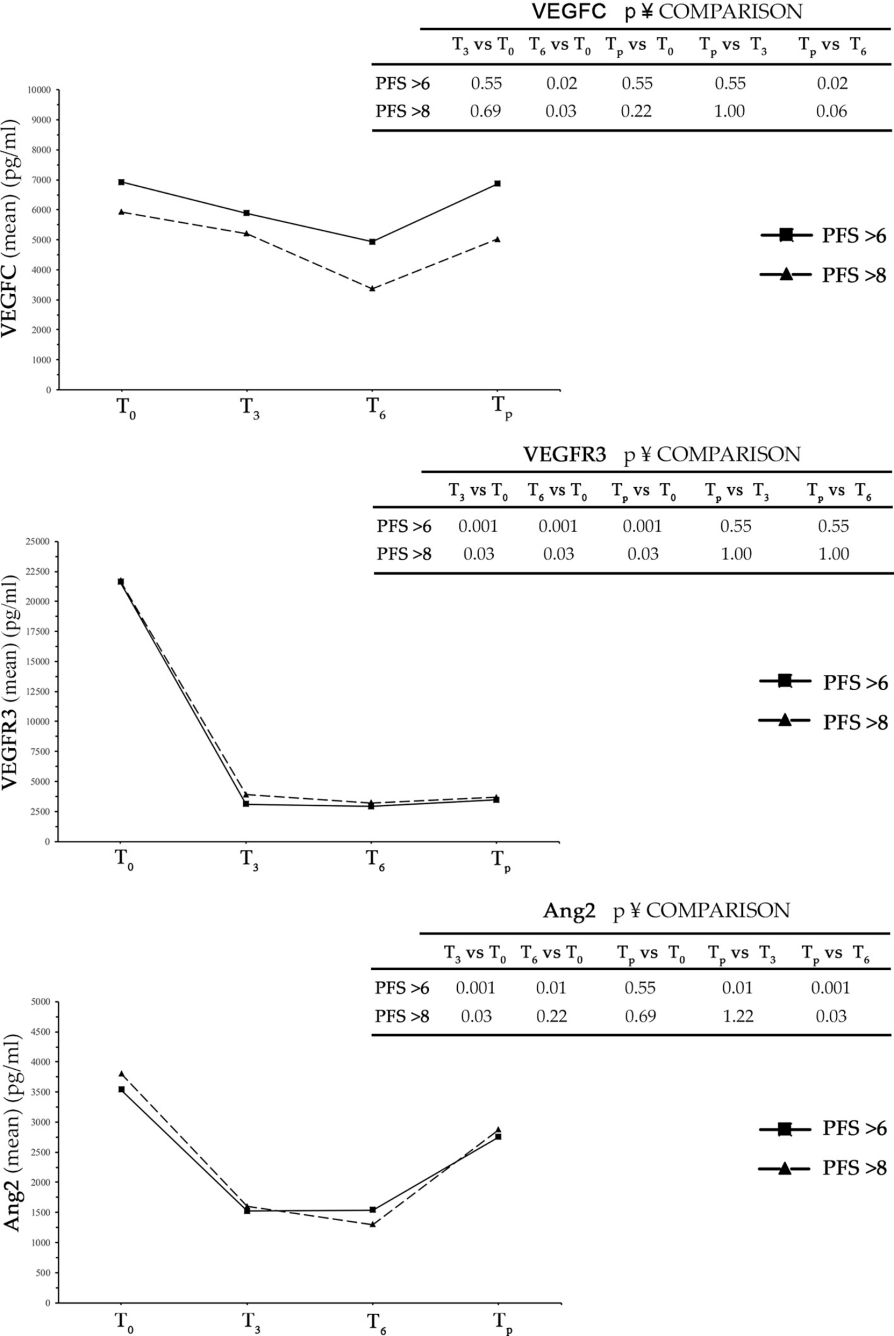
In the three graphs is shown trends over time of VEGFC, VEGFR3, and Ang2 in patients with PFS > 6 months and PFS > 8 months. PFS, progression-free survival.

In patients with control of disease after the first radiological evaluation (CD group), the levels of VEGFC, VEGFR3, and Ang2 were evaluated at T_0_, T_3_, T_6_, and the time of progression of disease (T_p_). In the analysis, the CD group was divided into two subgroups: one included patients with PFS > 6 months (n = 11) and the other patients with a PFS > 8 months (n = 6) (**Figure 1**).

In both subgroups, there was a reduction of all three analyzed markers from time T_0_ to T_3_. The decrease was highly pronounced in the case of VEGFR3 and Ang2, while in the case of VEGFC a relevant but non-significant reduction was observed. A further decrease was detected from time T_3_ to T_6_, to a higher extent in VEGFC and Ang2, with respect to VEGFR3. At the time of progression, all three analyzed angiogenetic factors showed an increase in serum level over T_6_ that was clearly evident for Ang2 and minor or insignificant for VEGFR3. Interestingly, while the decrease in serum levels detected for all three factors in intervals from T_0_ to T_3_ and from T_3_ to T_6_ was greater in the group of patients with PFS > 8 months compared to that with PFS > 6 months, the detected increase in the interval from T_6_ to T_p_ was PFS independent (**Figure 1** and **Table S2**).

To assess whether marker changes at time T_3_ were predictive for the therapeutic outcome, the univariate analysis was performed using the Cox model to calculate an HR of progression. The ΔT_0_–T_3_ values of each marker were divided into tertiles such that each comprised 33% of patients. The first tertile was considered as a reference (HR = 1). As shown in **Table 5** for VEGFC, all patients with ΔT_0_–T_3_ higher than 400 pg/ml had a probability for progression decreased by 25% compared to patients of the reference category. Considering the VEGFR3 receptor, in patients with ΔT_0_–T_3_ between 19,288.89 and 24,622.22 pg/ml, the probability of the disease progression was halved (51%) compared to that of the reference group. In contrast, when the VEGFR3 ΔT_0_–T_3_ was greater than 24,622.22 pg/ml, the probability for progression increased by 84%. In the case of Ang2, for ΔT_0_–T_3_ greater than 2,350 pg/ml, the risk of progression decreased significantly by 58% compared to the reference group (**Table 5**).

**Table 5:** Correlation between marker changes at time T_3_ and therapy outcome.

Biomarkers	HR	se (HR)	p	95% CI
**VEGFC (pg/ml)**				
**<−1,000 [Ref. Category]**	1			
**−1,000 to 400**	1.03	0.46	0.94	0.43–2.50
**≥ 400**	0.75	0.33	0.51	0.31–1.78
**VEGFR3 (pg/ml)**				
**<19,288.89 [Ref. Category]**	1			
**19,288.89–24,622.22**	0.49	0.23	0.13	0.20–1.24
**≥24,622.22**	1.84	0.80	0.16	0.79–4.30
**Ang2 (pg/ml)**				
**<892.86 [Ref. Category]**	1			
**892.86–2,350.00**	0.78	0.33	0.56	0.33–1.81
**≥2,350.00**	0.42	0.19	0.05	0.17–1.00

HR, hazard ratio; se(HR), standard error HR. CL, Confidential Interval.

It can be observed, in the graphs shown in **Figure S1**, how the cumulative hazard varied over time for each of the three tertiles. In order to find possible correlations between the biomarkers examined, a linear regression analysis of the basal levels of VEGFA, VEGFR2, VEGFC, and Ang2 was performed with respect to each of the markers examined. We found a slight but significant association of VEGFC with its receptor VEGFR3 (β = 0.19, se(β) = 0.08, p = 0.03, CI = 0.01–0.037, R^2^ = 0.26) and a slight but significant association of Ang2 with VEGFR3 (β = 0.09, se(β) = 0.03, p = 0.005, CI = 0.03–0.16, R^2^ = 0.42) (**Table S3**).

## Discussion

Tumor growth and progression rely on the tumor vascular network for the necessary supply of oxygen and nutrients. Tumor angiogenesis is the result of a program finely tuned by a plethora of growth factors, EC proliferation, extracellular matrix (ECM) remodeling, and stromal cell interactions. Several pro-angiogenic factors have been identified; the most important of them is represented by the VEGF family. Blockade of VEGFRs or ligands by neutralizing antibodies are among the most studied therapeutic approaches in preclinical and clinical research for inhibition of angiogenesis. Despite the promising results, the benefits of these therapeutic weapons are reduced due to the phenomena of induced or acquired resistance that is implemented through the activation of alternative angiogenic mechanisms that bypass the block exerted by specific inhibitors (23). The selection of patients who may benefit from a given therapeutic approach has been made possible in many cancers by the knowledge of biomarkers with predictive value. However, the availability of markers is very limited in the case of antiangiogenic therapy (21). The identification of biomarkers for antiangiogenic therapy in GC is even more complex due to the extreme molecular heterogeneity of this type of cancer (24).

The members of the VEGF family, including ligands and receptors, are studied as principal candidates for predictive/prognostic biomarkers, and their high serum levels have been associated with a poor prognosis of GC (25–28). To date, retrospective studies focused on the analyses of baseline levels of circulating markers have failed to identify biomarkers predictive for response to antiangiogenic therapy in advanced GC (17, 29). Therefore, more recent prospective studies have focused on changes in some circulating markers over time compared to baseline levels measured before initiation of therapy. In this line, the recent prospective study conducted by Van Cutsem and colleagues on patients from the RAINBOW cohort did not identify specific predictive biomarkers for response to treatment with ramucirumab and PTX. However, the authors found a trend of response in plasma levels of VEGFD, PLGF, and Ang2 during therapy. The plasma levels of VEGFD and PLGF increased from baseline during treatment and decreased after treatment suspension. Instead, Ang2 showed a decrease during treatment and increase upon treatment suspension (22).

In this framework, the present prospective study was aimed to investigate whether VEGFs and VEGFRs change during the pharmacological treatment and to identify possible correlations with clinical outcomes. Furthermore, the study was extended at some of the principal angiogenic factors known to be involved in ramucirumab resistance like PDGF, PIGF, and EGF including also angiogenesis modulators such as Ang2/sTIE-2 (17, 30, 31).

Thirty-five patients with advanced GC undergoing second-line therapy with ramucirumab and PTX were included in the study, and an analysis of selected angiogenic biomarkers levels by serum sampling over multiple time points was performed. The population, according to the response at the first radiological evaluation, was divided into “Control Disease Group” and “Progression Disease Group,” with median PFS of 8.8 and 2.8 months, respectively. In a first comparative analysis, the basal levels of biomarkers in the two groups were compared. This analysis revealed differences between the groups, although not statistically significant for the markers examined. The basal levels of VEGFA, VEGFC, PLGF, and Ang2 were higher in the CD group than in the PD group, whereas the basal levels of VEGFR2, VEGFR3, and sTie2 receptors were higher in the PD group.

To assess any differences between CD and PD patients, the levels of the different markers measured at baseline were compared with those measured at the beginning of the third cycle of therapy and with those found at the time of radiological and clinical disease progression.

The results showed that the levels of VEGFA, VEGFD, and PLGF ligands tend to increase significantly already during the first months of therapy. This result is explained by the displacement of the main VEGFR2 ligands, such as VEGFA, VEGFD, and PLGF, due to the ramucirumab binding. The trend of VEGFC was different in the two groups, with a decrease in its levels in the CD group and an increase in the PD group. As a result, the ΔT_0_–T_3_ of VEGFC was positive in the CD group (where the factor decreased by 9.2%) and negative in the PD group (where the factor increased by 31%). This finding is of particular interest since it could be related to an inhibition of the VEGFR3/VEGFC axis and thus of lymphangiogenesis. In contrast to VEGF ligands, VEGFR levels decreased during therapy. There was also a decrease in serum Ang2 levels during therapy, but the degree of this reduction was significantly greater in the CD group than in the PD group. Accordingly, the ΔT_0_–T_3_ value of Ang2 was significantly higher in the CD group than in the PD group.

It is widely accepted that Ang2 overexpression regulates vascular remodeling independently of VEGF, thus constituting a possible mechanism of acquired resistance during anti-VEGF therapies (32). A compromised vasculature leads to hypoxic conditions resulting in the production of signal-activating molecules that create a microenvironment with an immunosuppressive phenotype devoid of effector T cells. Therefore, VEGF and Ang2 can be considered not only as the main players in the angiogenic switch but also as powerful immune modulators (4, 5). Several studies demonstrated that upregulation of VEGF, through interaction with VEGFR2, is responsible for the Ang2 overexpression by ECs in the stroma surrounding the tumor. Moreover, it is well known that high levels of circulating Ang2 correlate with poor prognosis in several tumors. Results from the AVAGAST study showed that this factor could be considered as a prognostic marker in advanced GC treated with bevacizumab (30). The blockage of VEGFR2 due to ramucirumab binding could explain the observed Ang2 decrease (30, 32, 33).

In all patients examined, the analysis of serum levels of the other biomarkers did not show a strong pattern of their expression during treatment, and accordingly, they were not considered in subsequent comparative analyses. Patients in the CD group were followed up until progression disease. In this group of patients during treatment, a continuing increase in the levels of VEGFA, VEGFD, and PLGF was detected until progression disease. Conversely, the levels of VEGFC presented a rapid increment at the time of progression after the initial decrease. In addition, there was an increase in the levels of VEGFRs and Tie2 receptors and a further significant increase in the levels of Ang2. The extent of the changes in VEGFC and Ang2 over time was greater and more significant both when the analysis included time after the third cycle (ΔT_0_–T_6_) of therapy and when the analysis was restricted to a subgroup of patients with longer PFS (PFS > 8 months). The results described suggested a crucial role of the VEGFC/VEGFR3 and Ang2/Tie2 axes in determining response to therapy. Furthermore, the decrease in angiogenic markers such as Ang2 and VEGFC could be also related to the vessel “normalization window” and a permissive immune phenotype (4).

To assess the predictive value of the ΔT_0_–T_3_ of these markers, the univariate analysis using the Cox model was performed. The results of this analysis showed that a greater change in VEGFC and Ang2 levels measured at the beginning of the third cycle of therapy corresponded to a lower risk of progression and thus a longer PFS. In the case of Ang2, for ΔT_0_–T_3_ greater than 2,350 pg/ml, the risk of progression decreased significantly by 58% [HR = 0.42, se(HR) = 0.19; p = 0.05 (95% CI 0.17–1.00)]. Therefore, the ΔT_0_–T_3_ of Ang2 may be considered as an outcome predictor of ramucirumab–PTX therapy. The limitations of the study were related to the small number of patients recruited, which makes it difficult to stratify the analyses performed on the basis of parameters such as age, sex, and clinicopathological characteristics of the tumor. In addition, further studies are needed to establish possible correlations between changes in circulating biomarkers over time and their expression *in situ* in the tumor and surrounding microenvironment, since there are indications that the levels of these markers are associated with a different outcome depending on their expression site [17]. Unfortunately, the analyses did not show significant correlations between basal VEGFC and Ang2 levels, although slight but significant relationships between VEGFC and VEGFR3 and between Ang2 and VEGFR3 were detected.

Nevertheless, the rapid increase in VEGFC and Ang2 at the time of progression could suggest the activation of alternative pathways, represented by VEGFC/VEGFR3 and Ang2/Tie2 able to counteract the VEGFR2 blockade by ramucirumab. These results support the idea, already present in the literature, that there is a close correlation between angiogenesis and lymphangiogenesis and that this is crucial for tumor progression and spread. It is known that the upregulation of Ang2 by lymphatic ECs is induced by the action of VEGFC and its binding to VEGFR2 (34).

## Conclusions

This prospective study focused on the analysis of serum levels of angiogenic biomarkers in patients with advanced GC undergoing second-line therapy with ramucirumab and PTX. All VEGF family members as well as Ang2 and its receptor Tie2 were considered. Sera were sampled at each cycle of therapy until the time of progression. The aim of our study was to identify possible predictive markers and to evaluate whether variations in a given marker over time could be predictive for therapeutic outcomes. Overall results indicated that patients with longer PFS presented higher baseline levels of VEGFs and Ang2 compared to those with shorter ones. None of the baseline markers were found to be predictive for outcomes to therapy. However, the results clearly showed that a greater decrease in VEGFC and Ang2 levels measured at the beginning of the third cycle of therapy corresponded to a lower risk of progression and thus a longer PFS. Significantly, changes in Ang2 levels greater than or equal to 2,350 pg/ml decreased the risk of progression by 58%. In addition, there was a significant increase in VEGFC and Ang2 at the progression time, which could suggest the activation of alternative pathways such as VEGFC/VEGFR3 and Ang2/Tie2 that may counteract the blockade of VEGFR2 by ramucirumab. These findings support the rationale that dual inhibition of Ang2 and VEGFRs could increase the vessel normalization window. Furthermore, this combined blockade elicits antitumor immunity; therefore, cotargeting of angiogenesis and immune checkpoints could improve the efficacy of GC therapy (35).

## Data Availability Statement

The raw data supporting the conclusions of this article will be made available by the authors, without undue reservation.

## Ethics Statement

The studies involving human participants were reviewed and approved by the Ethics Committee of I.R.C.C.S “Giovanni Paolo II” Cancer Institute of Bari (Italy), n°139/c.e. 28-06-2017. The patients/participants provided their written informed consent to participate in this study.

## Author Contributions

Conceptualization: RD’A, MR, and CL. Methodology: RD’A, MR, AS, and GL. Software: RD and VG. Validation: RD, CM, and CL. Formal analysis: RD’A, MR, and VG. Investigation: RD’A, MR, AS, and GL. Resources: GG, CM, IL, and CL. Data curation: RD’A, RD, VG, and CL. Writing—original draft preparation: RD and CL. Writing—review and editing: ML, FV, GG, CM, and CL; visualization, RD’A, MR, AS, and CM; supervision, GG, CM, FV, and CL. Project administration: GG, CM, and CL. Funding acquisition: CM. All authors have read and agreed to the published version of the manuscript.

## Funding

This research was funded by the Italian Ministry of Public Health (n.7/2017).

## Conflict of Interest

The authors declare that the research was conducted in the absence of any commercial or financial relationships that could be construed as a potential conflict of interest.

## Publisher’s Note

All claims expressed in this article are solely those of the authors and do not necessarily represent those of their affiliated organizations, or those of the publisher, the editors and the reviewers. Any product that may be evaluated in this article, or claim that may be made by its manufacturer, is not guaranteed or endorsed by the publisher.
