# Corrigendum: Variations in circulating levels of angiopoietin-2 over time are predictive of ramucirumab–paclitaxel therapy outcome in advanced gastric cancer: results of prospective study

**DOI:** 10.3389/fonc.2023.1187014

**Published:** 2023-04-14

**Authors:** Rosalba D’Alessandro, Maria Grazia Refolo, Annalisa Schirizzi, Giampiero De Leonardis, Rossella Donghia, Vito Guerra, Gianluigi Giannelli, Ivan Roberto Lolli, Maria Maddalena Laterza, Ferdinando De Vita, Caterina Messa, Claudio Lotesoriere

**Affiliations:** ^1^ Laboratory of Experimental Oncology, National Institute of Gastroenterology - IRCCS “Saverio de Bellis”, Castellana Grotte, Italy; ^2^ Data Science Unit, National Institute of Gastroenterology - IRCCS “Saverio de Bellis”, Castellana Grotte, Italy; ^3^ Scientific Direction, National Institute of Gastroenterology - IRCCS “Saverio de Bellis”, Castellana Grotte, Italy; ^4^ Medical Oncology Unit, National Institute of Gastroenterology - IRCCS “Saverio de Bellis”, Castellana Grotte, Italy; ^5^ Complex Operating Unit Oncologia, Local Health Authority Napoli 2 Nord, P.O. “S.M. delle Grazie”, Naples, Italy; ^6^ Division of Medical Oncology, Department of Precision Medicine, School of Medicine, University of Study of Campania “Luigi Vanvitelli”, Naples, Italy

**Keywords:** angiogenesis, gastric cancer, target therapy, biomarkers, cancer progression

In the published article, there were errors in the author affiliations list. Instead of

“^1^Laboratory of Experimental Oncology, National Institute of Gastroenterology, “Saverio de Bellis” Research Hospital, Castellana Grotte, Italy; ^2^National Institute of Gastroenterology, “Saverio de Bellis” Research Hospital, Castellana Grotte, Italy; ^3^Scientific Direction, National Institute of Gastroenterology “Saverio de Bellis”, Research Hospital, Castellana Grotte, Italy; ^4^Medical Oncology Unit, National Institute of Gastroenterology, “Saverio de Bellis” Research Hospital, Castellana Grotte, Italy; ^5^Complex Operating Unit Oncologia, Local Health Authority Napoli 2 Nord, P.O. “S.M. delle Grazie”, Naples, Italy; ^6^Division of Medical Oncology, Department of Precision Medicine, School of Medicine, University of Study of Campania “Luigi Vanvitelli”, Naples, Italy”, the author list should have been “^1^Laboratory of Experimental Oncology, National Institute of Gastroenterology - IRCCS “Saverio de Bellis”, Castellana Grotte, Italy; ^2^Data Science Unit, National Institute of Gastroenterology - IRCCS “Saverio de Bellis”, Castellana Grotte, Italy; ^3^Scientific Direction, National Institute of Gastroenterology - IRCCS “Saverio de Bellis”, Castellana Grotte, Italy; ^4^Medical Oncology Unit, National Institute of Gastroenterology - IRCCS “Saverio de Bellis”, Castellana Grotte, Italy; ^5^Complex Operating Unit Oncologia, Local Health Authority Napoli 2 Nord, P.O. “S.M. delle Grazie”, Naples, Italy; ^6^Division of Medical Oncology, Department of Precision Medicine, School of Medicine, University of Study of Campania “Luigi Vanvitelli”, Naples, Italy”.

The authors apologize for this error and state that this does not change the scientific conclusions of the article in any way. The original article has been updated.

