# Supplementary material for: Variations in Circulating Levels of Angiopoietin-2 Over Time Are Predictive of Ramucirumab–Paclitaxel Therapy Outcome in Advanced Gastric Cancer: Results of Prospective Study
Source: Front Oncol. 2022 Apr 6;12:862116. doi: 10.3389/fonc.2022.862116 (PMC9019360; doi:10.3389/fonc.2022.862116)
Supplement: Supplementary file 1 [file DataSheet_1.pdf]

Supplementary Material

1 Supplementary Figures and Tables

1.1 Supplementary Figure

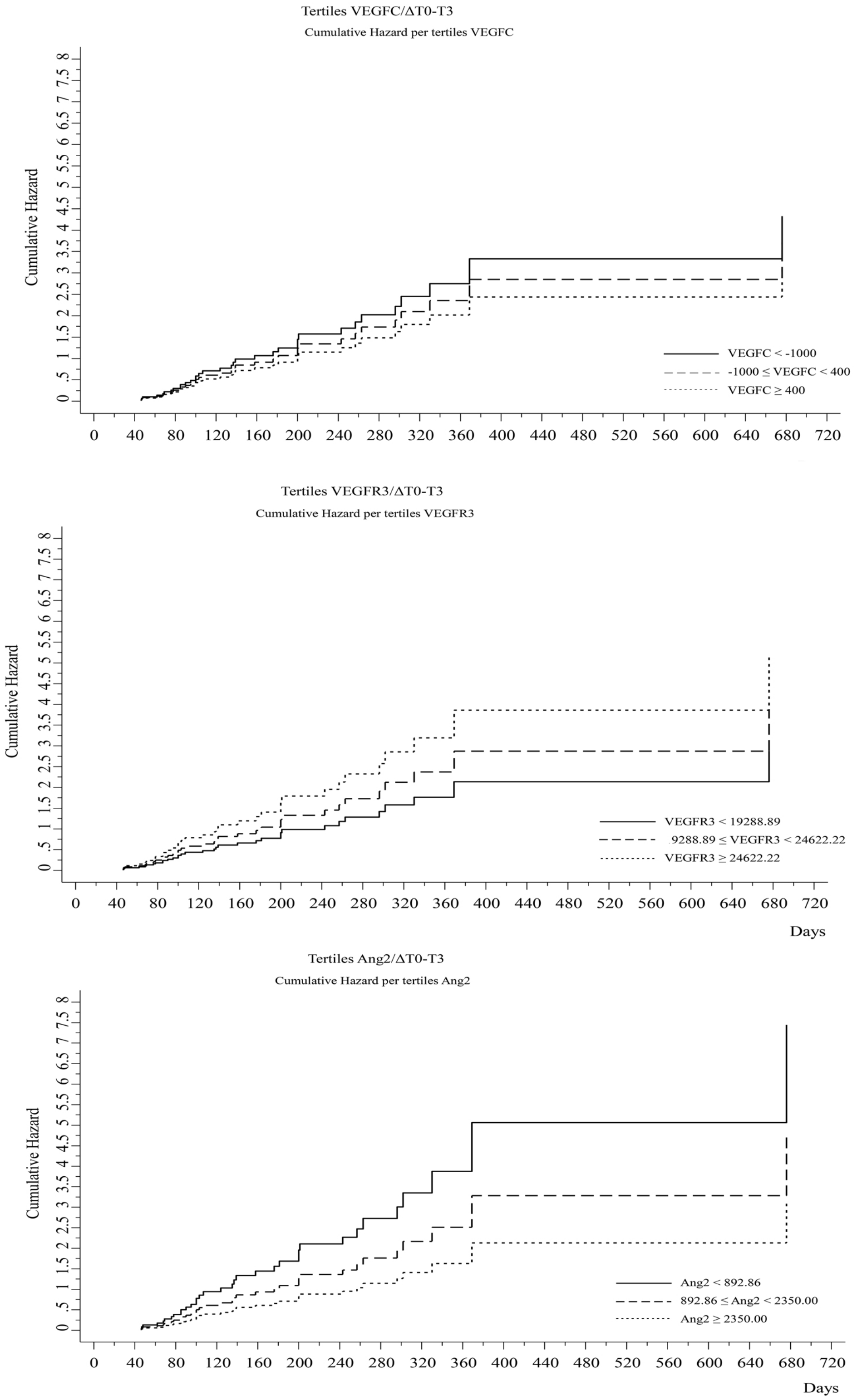

FIGURE 1S In these three graphs is shown how the cumulative hazard varies over time for each of the three tertiles of VEGFC, VEGFR3 and Ang2 $\Delta_{T0\text{-}T3}$

1.2 Supplementary Tables

*Biomarkers	Δ_{T0-T3} CD patients (n=17)	PD patients (n=18)	p^
**VEGFC#**	590.29±1732.78	-1687.01±2898.69	0.02
**Ang2#**	2018.43±1209.89	920.96±1892.42	0.05
**PLGF**	-29.87±31.52	-33.76±24.16	0.41
**VEGFD**	-264.51±168.37	-181.57±162.14	0.30
**VEGFA**	-240.38±234.89	-290.93±168.11	0.26
**sVEGFR1**	16.73±83.97	-2.44±9.49	0.41
**sVEGFR2**	109.61±704.49	335.98±884.00	0.36
**VEGFR3#**	20138.10±8857.56	24898.74±11633.47	0.17
**sTie2**	585.91±827.08	541.21±1343.28	0.52

TABLE 1S Overall difference between serum biomarker levels at T_3 and T_0 time in two groups

* Concentration *pg/ml* Mean±DS;

^ Wilcoxon rank-sum (Mann–Whitney) test

Detected with uniplex ELISA assay

Abbreviation: CD, Control Disease; PD, Progression Disease; Δ_{T0-T3}, T_0-T_3 delta

frontiers

A	CD patients PFS >6 (n=11)								
*Biomarkers	Basal levels (T_0)	3° Cycle (T_3)	6° Cycle (T_6)	time of progression (T_p)[§]	p [¥]				
	(a)	(b)	(c)	(d)	(b)vs(a)	(c)vs(a)	(d)vs(a)	(d)vs(b)	(d)vs(c)
VEGFC[#]	6923.41±4617.13	5889.92±3426.10	4939.17±3841.39	6870.98±5044.01	0.55	0.02	0.55	0.55	0.02
Ang2[#]	3535.80±1807.91	1522.29±740.11	1538.87±754.14	2750.69±607.66	0.001	0.01	0.55	0.01	0.001
VEGFR3[#]	21629.74±10414.25	3108.85±2470.40	2913.79±2235.08	3448.36±3040.71	0.001	0.001	0.001	0.55	0.55

B	CD patients PFS > 8 (n=6)								
*Biomarkers	Basal levels (T_0)	3° Cycle (T_3)	6° Cycle (T_6)	time of progression (T_p)[§]	p [¥]				
	(a)	(b)	(c)	(d)	(b)vs(a)	(c)vs(a)	(d)vs(a)	(d)vs(b)	(d)vs(c)
VEGFC[#]	5924.17±2955.78	5207.50±1886.22	3372.78±1773.59	5021.39±1512.66	0.69	0.03	0.22	1.00	0.06
Ang2[#]	3804.21±2095.45	1601.03±962.09	1298.65±651.40	2872.26±333.15	0.03	0.22	0.69	0.22	0.03
VEGFR3[#]	21731.49±12258.69	3904.70±3011.02	3223.90±2658.97	3672.32±3665.01	0.03	0.03	0.03	1.00	0.03

TABLE 2S Trend over time of VEGFC, VEGFR3 and Ang2 in patients with PFS >6 months (A) and PFS >8 months (B)

[*] Concentration pg/ml Mean±DS; [¥] Sign test

[§] 1 patient undergoing therapy; [#] Detected with uniplex ELISA assay

Abbreviation: CD, Control Desease; PFS, Progression Free Survival

Biomarkers	VEGFC β	VEGFC se (β)	VEGFC p	VEGFC 95% C.I.	VEGFC R^2	Ang2 β	Ang2 se (β)	Ang2 p	Ang2 95% C.I.	Ang2 R^2
**VEGFC**	N/A	N/A	N/A	N/A	N/A	0.07	0.10	0.49	-0.14 to 0.28	0.03
**Ang2**	0.47	0.66	0.49	-0.93 to 1.87	0.03	N/A	N/A	N/A	N/A	N/A
**PLGF**	-78.54	121.18	0.53	-336.83 to 179.74	0.03	-71.54	43.80	0.12	-164.89 to 21.82	0.15
**VEGFD**	4.78	8.04	0.56	-12.35 to 21.91	0.02	-1.61	3.12	0.61	-8.25 to 5.04	0.02
**VEGFA**	4.83	8.22	0.56	-12.68 to 22.35	0.02	1.55	3.19	0.63	-5.25 to 8.35	0.01
**sVEGFR1**	-0.58	12.97	0.96	-28.22 to 27.06	0.0001	7.29	4.65	0.14	-2.62 to 17.21	0.14
**sVEGFR2**	1.73	1.21	0.17	-0.85 to 4.30	0.12	0.33	0.49	0.51	-0.71 to 1.38	0.03
**VEGFR3**	0.19	0.08	0.03	0.01 to 0.37	0.26	0.09	0.03	0.005	0.03 to 0.16	0.42
**sTie2**	0.61	0.84	0.48	-1.19 to 2.41	0.03	-0.05	0.33	0.89	-0.76 to 0.66	0.001

TABLE 3S Linear regression model of VEGFC or Ang2 on each biomarker at baseline in patients with disease control

Abbreviation: CI, Confidence Interval
